# Pregnancies, intentions, and fertility behaviors during use of the Creighton Model FertilityCare System after initial intention to avoid pregnancy: Results from the Creighton Model effectiveness, intentions, behaviors assessment study

**DOI:** 10.1371/journal.pone.0328806

**Published:** 2025-07-29

**Authors:** Joseph B. Stanford, Shahpar Najmabadi, Chun-Pin Esther Chang, Daniel Opoku Agyemang, Kathy Rivet, Christina A. Porucznik

**Affiliations:** 1 Department of Family and Preventive Medicine, Office of Cooperative Reproductive Health, Division of Public Health, Spencer Fox Eccles School of Medicine, University of Utah, Salt Lake City, United States of America; 2 Marguerite d’Youville FertilityCare Services, Manchester, New Hampshire, United States of America; Mizan-Tepi University, ETHIOPIA

## Abstract

**Background:**

Knowledge of the fertile and infertile phases of the menstrual cycle can be applied to conceive or to avoid pregnancy. Fertility intentions and sexual behaviors during the fertile time may influence whether and when pregnancy occurs. The Creighton Model FertilityCare System (CrMS) is a specific system of fertility appreciation used to conceive or to avoid pregnancy. The objective of this paper is to report intentions, behaviors, and pregnancy rates during use of the CrMS among couples who initially intended to avoid pregnancy.

**Data and methods:**

We analyzed a prospective cohort study conducted in 17 CrMS centers across the USA and Canada, following 296 couples for up to one year after onset of initial use of the CrMS to avoid pregnancy. Baseline data included demographics, motivations, and pregnancy intentions for each partner. Couples contributed 2894 menstrual cycles, most of which had data collected (by questionnaires and daily diary) on cycle-specific pregnancy intentions, days of potential fertility, and fertility behaviors. Pregnancies were prospectively actively ascertained.

**Results:**

We found a high concordance (91%) in cycle pregnancy intentions between partners. However, 44% of cycles with strong intentions to avoid pregnancy included intercourse on potentially fertile days or days of undetermined fertility status. Across all sensitivity scenarios, cumulative 13-cycle pregnancy rates with cycle intention to conceive ranged from 88.0% to 89.8%, and cumulative 13-cycle pregnancy rates with cycle intention to avoid ranged from 29.1% to 35.3%. In multivariate analysis, baseline motivations and intentions for pregnancy within 2 years were strongly correlated with the likelihood of pregnancy, more so than cycle intentions.

**Conclusion:**

The findings suggest that in some populations using natural family planning, baseline motivations and intentions may be more strongly related to pregnancy rates than cycle intentions. Our findings also highlight essential elements for evaluating correct use, including complete recording of intercourse and its timing.

## Introduction

Methods of family planning based upon identifying the days of potential fertility in females, known as natural family planning (NFP) or fertility-awareness based methods (FABM), identify days in the menstrual cycle when intercourse is likely to result in pregnancy, using various biomarkers [1,2]. With this information, couples have the opportunity to align their sexual behavior with the desired outcome of avoiding pregnancy or conceiving [3–5]. Most long-term users of NFP or FABM methods will spend some of their time using the method to avoid pregnancy, and some of their time using it to conceive, which implies that they will also transition between these intentions [6,7].

The Creighton Model FertilityCare System (CrMS) is a method of natural family planning, fertility appreciation, and woman’s health assessment and promotion [8]. It was first developed based on initial research at St. Louis University into the Billings Ovulation Method, and further developed and implemented by Dr. Hilgers and colleagues at the Creighton University Medical Center and the Saint Paul VI Institute for the Study of Human Reproduction, Omaha, Nebraska [9–11]. It is based on standardized observation and recording of vaginal discharge (especially from cervical mucus) and vaginal bleeding, interpretation of these biomarkers for fertility and health status, and teaching by trained teachers using a case management approach [12].

The instruction of the CrMS emphasizes the versatility of the method to avoid pregnancy or to conceive at any point in time, as described by the term, fertility appreciation [13]. Thus, assessing the effectiveness of the CrMS requires some type of assessment of how the method is being used by a given couple at any given point in time [14]. Prior effectiveness studies of the CrMS used to avoid pregnancy have included detailed assessments of fertility tracking and sexual behaviors associated with each pregnancy [15–18]. These studies used a standardized detailed pregnancy evaluation, which is an integral component of the CrMS, with the expectation that a pregnancy evaluation should be completed for every pregnancy [19]. However, prior studies have not reported prospective measures of intention and behavior across all cycles, and lacked detailed information about the fertility tracking and sexual behaviors of users who did not become pregnant [20,21].

The study of Creighton Model Effectiveness, Intentions, Behaviors Assessment (CEIBA) was conducted for the purpose of assessing fertility motivations, intentions, fertility-related sexual behaviors, and their impact on effectiveness of the CrM to avoid pregnancy and to conceive. We have previously reported on the demographic characteristics and the baseline childbearing motivations, desires and intentions of females and males (heterosexual couples) who had enrolled in CEIBA and started use of the CrM with the initial intention to avoid pregnancy [21]. The purpose of this paper is to report on prospectively assessed pregnancy intentions and fertility behaviors over time (i.e., sexual intercourse on days identified as fertile or non-fertile), and to report pregnancy rates during use of the CrMS. We analyzed pregnancy rates in several different ways: by prospectively assessed pregnancy intentions [22], by correct use to conceive [23], and by correct use to avoid (analogous to “perfect use”) [24]. We also investigate user characteristics, motivations, and intentions associated with the likelihood of pregnancy. Finally, we report pregnancy rates according to the CrMS pregnancy classification system and lifetable analysis to compare with prior CrMS studies [14,16].

## Materials and methods

The CEIBA study was a prospective cohort study conducted in 17 Creighton Model FertilityCare Centers throughout the United States and Canada. We have previously reported enrollment and conduct of the study [21]. Briefly, couples were eligible for the study if they were learning the CrMS for the first time with the initial intention to avoid pregnancy, or entering instruction after a gap of at least 6 months without using the CrMS (such as after a pregnancy). Females were required to be between the ages of 18 and 39, inclusively, and the couple could not have any identified history, circumstance, or current treatment that would reduce fertility. Based on logistical and budget considerations, the original enrollment target was 300 couples. Couples attended a CrMS introductory session, and individual follow-up instructional sessions for up to 8 additional sessions in the first year of use [25]. To be included in this analysis, couples needed to attend at least one follow-up session within 6 weeks of the introductory session (usually scheduled at 2 weeks after the introductory session).

### Research ethics approval

Research ethics approval was obtained through the University of Utah Institutional Review Board, IRB #00034487, with the initial approval on July 6, 2009. Both the female and the male partner received the information about the study, and each signed a written informed consent document.

### Study entrance and exit

The study began enrollment on September 22, 2009 and closed enrollment on December 3, 2011. Both the female and the male partner received the information about the study and signed the informed consent document. Once a couple signed the informed consent document, they were entered into the study. Couples also completed an entrance questionnaire that included questions about intentions and motivations for childbearing in the future. Some couples in the study were not sexually active until up to 6 months after the consent date. The analytic start date for this analysis was either start date of cycle during which consent occurred, or the start date of the cycle with the first recorded sexual intercourse, whichever came later. Couples were followed actively for up to one year by both the CrMS teacher and the study research staff. Couples exited the study by completing one year without pregnancy, achieving a pregnancy, being lost to follow-up, or for other reasons.

### CrMS chart (daily diary)

In the standard teaching of the CrMS, couples are taught to track and record observations of vaginal bleeding, and vaginal discharge (including cervical fluid) on a daily basis on the CrMS chart (a daily diary), using the standardized vaginal discharge recording system [26]. They also are asked to record sexual intercourse for each day. Information from this chart was used to interpret each day as fertile, non-fertile, or of unknown fertility (i.e., missing information), and the timing of acts of sexual intercourse on fertile or non-fertile days of the cycle. A more detailed description of the CrMS charting and the identification of days as fertile, non-fertile, or of unknown fertility is provided in the S1 Text.

### Prospective assessment of pregnancy intentions

A brief electronic questionnaire was sent by weblink or text message at the start of each cycle (SOC questionnaire) to each partner separately, which asked about intentions for pregnancy in the *upcoming cycle*, using the following scaled response options: we are trying as hard as possible to get pregnant; we are trying to get pregnant; we are neither trying to get pregnant nor are we trying to avoid getting pregnant; we are trying to avoid getting pregnant; we are trying as hard as possible to avoid getting pregnant; we plan to completely abstain from all genital contact during this cycle. Another question asked whether any other method was used in addition to CrMS in the *cycle immediately prior* to the questionnaire (such as basal body temperature, urine hormone testing, condoms, or withdrawal). The SOC questionnaire was sent out at the time of the expected menses for each cycle, based on prior cycle history.

We evaluated the SOC pregnancy intentions descriptively for concordance between the female and the male partner for each cycle. We also described the correlation between pregnancy intentions and fertility behaviors, i.e., timing of intercourse on fertile or non-fertile days, or days of unknown fertility. For these analyses, if there was a discrepancy between the female and the male participant, the “most achieving” response was used.

Sometimes, the SOC questionnaire was filled out later than it was requested. Our primary analyses included only those SOC questionnaires filled out within the first 11 days of the cycle, to allow for prospective assessment of intentions, since day 11 is usually before ovulation [27]. We also conducted secondary analyses including all SOC questionnaires, including those filled out later than the first 11 days of the cycle.

### CrMS teacher assessments

During the CrMS follow-up teaching sessions, teachers assess the understanding and consistency of use of the CrMS for the woman and couple. The teachers record their assessments in a standardized form on the CrMS follow-up form. Key variables from these assessments were abstracted and assigned to each cycle based on the closest match between the visit and the cycle end date. These variables were used to assess cycles of correct use, described further below.

### Exit questionnaire

All couples who completed the study with or without pregnancy were asked to complete a study exit questionnaire (one for the woman and one for the man). For analyses of correct use, we included a question from the exit questionnaire about whether the couple had consistently recorded each act of intercourse during the study.

### Detection of pregnancy

We conducted active surveillance for pregnancy each cycle during the study, based on any lengthened postpeak phase (indicator of luteal phase), delay of expected timing of menses at beginning of next cycle (tracked with the monthly SOC questionnaires), and any other report from the couple or the teacher. For any possibility of pregnancy, the results of home or office pregnancy testing were solicited by direct contact with the participant.

### Analytic dataset for cycle-based analyses

**Fig 1:**
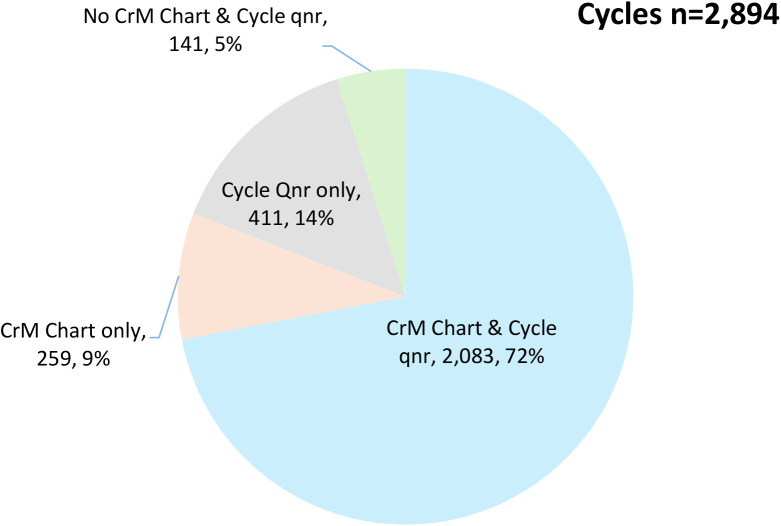
Sources of information for cycles in the final data set. Number and proportion of cycles with data from Creighton Model charts (CrM), start-of-cycle questionnaires (Cycle Qnr), both sources, or neither source.

We compiled a database of all menstrual cycles within the study period for each couple (2894 cycles). The first cycle contained the analytic start date, and the final cycle contained the exit date, as defined above, under *Study entrance and exit*. We had two sources of cycle information: the CrMS chart (daily diary), and the SOC questionnaires. We had complete information from both sources for 2083 (72%) of cycles. We had CrMS chart information only for 259 (9%) cycles, and SOC questionnaire information only for 411 cycles (5%; see **Fig 1**). We imputed cycles for which we had no other information, based on other available cycle lengths for the woman, 141 cycles (5%).

### Life table pregnancy probabilities by prospectively stated pregnancy intentions

We calculated cumulative pregnancy probabilities by life table over 13 cycles according to prospectively stated pregnancy intentions, either to conceive or to avoid pregnancy. For these analyses, being undecided was classified with trying to conceive, because the rates of pregnancy in these intention categories was very similar. We conducted sensitivity analyses excluding or including cycles with any identified use of condoms and withdrawal. (Details about identifying condom and withdrawal use are in the S1 Text.) We also used Cox proportional regression models to assess factors associated with the incidence of pregnancy in the study (i.e., hazard ratios).

### Cycles of correct use to conceive or to avoid pregnancy, and associated pregnancy probabilities

We identified cycles of correct use in three levels of analyses with progressively restrictive definitions. In all baseline analyses of correct use, we only included couples where at least one person had indicated on the exit questionnaire that they had recorded every act of intercourse and genital contact on their CrMS chart “all of the time without exception” or “nearly all of the time with maybe a rare exception.” We also required a complete CrMS chart with daily observations for the entire cycle, with at least one act of intercourse. At the next level, we further restricted the analysis to include only cycles where the CrMS teacher assessment had certified consistent observations, appropriate identification of the peak day, and accurate charting. At a third, most restrictive level, we further excluded cycles with any identified use of condoms or withdrawal, or nonstandard CrMS chart formats.

For correct use to conceive, we analyzed only cycles with any intercourse on a fertile day; or more restrictively, intercourse on a peak-type (estrogenic) mucus day, which is a more specific indicator of potential fertility [28,29]. For correct use to avoid pregnancy, we analyzed only cycles with no identified intercourse on any fertile day, nor on any day of unknown fertility. In further sensitivity analyses, we analyzed only cycles with intercourse only after Peak day (after estimated ovulation), or separately, only cycles with intercourse on the “last non-fertile day” before the peak day.

### Creighton Model pregnancy classification

We encouraged every participant who became pregnant to complete a pregnancy evaluation with their CrMS teacher. For the purposes of this study, all pregnancies were also reviewed again by an expert panel of CrMS educators and a medical consultant (K.D. Daly, RN, CFCE, K. Rivet, BS, CFCE and J.B. Stanford, MD, CFCMC). We used the standard CrMS pregnancy classification system [14,16]. Further details about the CrMS pregnancy classification are given in the S1 Text.

### Creighton Model life table pregnancy probabilities

We calculated pregnancy probabilities with CrMS, exactly as was done with prior CrMS studies [15–18]. These calculations are based on a multiple decrement life table that uses ordinal months (rather than cycles) [14]. Each couple entered the life table on the date they consented or first recorded sexual activity (whichever came later), and exited the life table with pregnancy (the outcome of interest), one year of use of the CrMS, stopping use of the CrMS for any reason, or being lost to follow-up. To allow direct comparability with prior CrMS studies, the same denominator of all months was used for all outcomes, i.e., there was no separation of months into avoiding-related or achieving-related use. In these calculations, the method-related pregnancy rate is not comparable to (and is lower than) the correct use pregnancy rate [24].

### Underlying reasons for CrMS pregnancy classification

After the initial expert review to determine the study pregnancy classification, the same expert team reviewed each pregnancy one more time to identify the key underlying reasons that each pregnancy was classified into a specific CrMS category. There were three guiding questions for this review:

1) What factors reasonably contributed to the pregnancy happening?
2) What factors supported the pregnancy classification?
3) Was there a behavior or attitude that contributed to the pregnancy happening?

Based on this review, we tabulated and summarized the underlying reason(s) for classifying each pregnancy into its specific CrMS pregnancy type. One pregnancy could have multiple reasons.

## Results

As we have reported previously, 1,132 new or returning couples were potentially eligible, of which 1,090 couples were screened, 429 were found eligible, and 305 female and 290 male participants signed the informed consent to enter the study. The reasons for not participating were detailed in our prior publication [21]. Of the 305 couples, five couples were subsequently found to be ineligible for the study entrance criteria, and four couples never attended a CrMS follow-up session, leaving 296 couples included in this analysis for this paper. The mean age of the female participants was 27.1 years, and of males 28.9 years. Most participants were married (51%) or engaged (40%), college graduates (females 79%, males 70%), and most (72%) females had no prior live births. About half (52%) of females wanted to have a child within 2 years (19% within one year); and 42% of males wanted to have a child within 2 year (8% within one year). See **Table 1**. Reasons for study completion or discontinuation are given in **Table 2**. There were 122 pregnancies within the study time period.

**Table 1:** Characteristics of CEIBA study participants.

	Female (N = 296)	Male(N = 296)
	N (%)	N (%)
**Age (years)**		
<30	222 (75.0)	183 (61.8)
<20	2 (0.7)	0 (0.0)
20–24	92 (31.1)	61 (20.6)
25–29	128 (43.2)	122 (41.2)
≥30	74 (25.0)	113 (38.2)
30–34	53 (17.9)	71 (24.0)
≥35	21 (7.1)	42 (14.2)
Missing	0 (0.0)	0 (0.0)
Range	19–40	20–54
Interquartile range	24–30	25–32
Median	26	28
Mean (standard deviation)	27.1 (4.5)	28.9 (5.6)
**Race and ethnicity**		
White (non-Hispanic)	241 (81.4)	250 (84.5)
Hispanic/Latino	22 (7.4)	24 (8.1)
Other	25 (8.5)	13 (4.4)
Missing	8 (2.7)	9 (3.0)
**Religion**		
Catholic	242 (81.8)	213 (72.0)
Protestant	26 (8.8)	45 (15.2)
Other	13 (4.4)	22 (7.4)
None	6 (2.0)	6 (2.0)
Missing	9 (3.0)	10 (3.4)
**Importance of religion in your life**		
Very important or important	231 (78.0)	195 (65.9)
Mixed	13 (4.4)	23 (7.8)
Not important, or prefer not to answer	4 (1.4)	13 (4.4)
Missing	48 (16.2)	65 (22.0)
**Marital status**		
Single	18 (6.1)	16 (5.4)
Engaged	118 (39.9)	118 (39.9)
Married	152 (51.4)	152 (51.4)
Divorced/Widowed/Separated	0 (0.0)	2 (0.7)
Missing	8 (2.7)	8 (2.7)
**Completed education**		
High School or Vocational School graduate or less	9 (3.0)	26 (8.8)
Some college	43 (14.5)	51 (17.2)
College graduate or more	234 (79.1)	208 (70.3)
Missing	10 (3.4)	11 (3.7)
**Occupational status**		
Professional	147 (49.7)	163 (55.1)
Technical/ Skilled & Unskilled laborer	19 (6.4)	43 (14.5)
Clerical/Sales	13 (4.4)	13 (4.4)
Homemaker	35 (11.8)	3 (1.0)
Student	60 (20.3)	43 (14.5)
Other	12 (4.1)	21 (7.1)
Missing	10 (3.4)	10 (3.4)
**Employed**		
Yes	220 (74.3)	258 (87.2)
No	67 (22.6)	30 (10.1)
Missing	9 (3.0)	8 (2.7)
**Household income relative to household federal poverty level, according to year of consent**		
<150%	35 (11.8)	
150-200%	16 (5.4)	
>200%	214 (72.3)	
Missing	31 (10.5)	
**Number of prior live births**		
None	213 (72%)	
1 or more	76 (25.7)	
Missing	7 (2.4)	
**Future number of children intended** ^*^		
None	23 (7.8)	21 (7.1)
1	26 (8.8)	21 (7.1)
2–4	159 (53.7)	148 (50.0)
≥5	26 (8.8)	23 (7.8)
Missing	62 (21.0)	83 (28.0)
**How soon want to have another child**		
No more children wanted	17 (5.7)	20 (6.8)
<1 year	55 (18.6)	25 (8.4)
1 year to <2 years	99 (33.4)	100 (33.8)
≥2 years	73 (24.7)	82 (27.7)
Unknown	4 (1.4)	5 (1.7)
Missing	48 (16.2)	64 (21.6)
**Positive childbearing motivation score (**higher score = more motivated to have a child)		
Range	1.3–4.0	1.0–4.0
Interquartile range	3.0–3.7	3.0–3.6
Median	3.4	3.4
Mean (standard deviation)	3.3 (0.5)	3.3 (0.5)
Missing	18	33
**Negative childbearing motivation score (**higher score = less motivated to have a child)		
Range		
Interquartile range	1.0–4.0	1.0–4.0
Median	1.7–2.5	2.0–2.7
Mean (standard deviation)	2	2.3
Missing	2.1 (0.6)	2.3 (0.6)
	18	35
**Prior use of Creighton Model FertilityCare System**		
Never used the CrMS	268 (90.5)	
Prior use of the CrMS^**^	27 (9.1)	
Missing	1 (0.3)	

* Total number of children intended minus current number of children; negative values assigned to zero.

** Prior use of the CrMS was either for gynecologic health only (not family planning), or at least 6 months ago for family planning.

**Table 2:** Final status of all couples at the end of the study.

Completion status	N	%
Completed study year without pregnancy	111	37.5
Pregnant	122	41.2
Unable to contact or nonresponsive for follow-up	27	9.1
Stopped using Creighton Model^*^	26	8.8
Separation or end of relationship	4	1.4
Refused further follow-up	3	1.0
No sexual activity	1	0.3
Other	2	0.7
**Total**	296	100.0

* Of the couples stopping the use of the Creighton Model, two started using a different method of natural family planning, and seven started using another method of family planning or birth control.

Females contributed 2894 cycles within the study (mean of 9.8 cycles each), with the details of available sources of information for each cycle shown in **Fig 1**. Out of 81,528 days in the data that had CrMS chart data available (2342 cycles), 33,567 days (41.2%) were identified as fertile, 40,888 (50.2%) days as non-fertile, and 7073 days (8.7%) of unknown fertility.

Across each cycle, there was a high concordance (91.3%) of pregnancy intentions between each female and male within each couple, as shown in Table 3. However, pregnancy intentions were not fully correlated with fertility behaviors. In **Table 4**, we report the proportion of cycles with intercourse on at least one day of fertility, according to the couple’s reported intention for the cycle. If one partner differed from the other for the cycle intention, we used the “most achieving” intention reported. Among cycles where at least one partner reported they were trying as hard as possible to conceive, there were 80% with intercourse on at least one day of known fertility. Among cycles where the intention was trying as hard as possible to avoid pregnancy, there were 29% with intercourse on at least one day of known fertility. Among cycles where the couple intention was to abstain from sexual intercourse, 5% of cycles included intercourse on at least one day of known fertility. The corresponding percentages were somewhat higher when the day of intercourse could be either on a day of known fertility or a day of undetermined fertility, e.g., among those trying as hard as possible to avoid pregnancy, 44% had intercourse on either a day of known fertility or undetermined fertility status.

**Table 3:** Pregnancy intentions of female and male (in couple) by each cycle (N = 2013 cycles)*.

Female, N (%)	Male, N (%)				
	**Achieving**	**Neutral**	**Avoiding**	**Abstaining**
**Achieving**	**69 (3.4)**	18 (0.9)	17 (0.8)	1 (0.05)
**Neutral**	4 (0.2)	**65 (3.2)**	27 (1.3)	1 (0.05)
**Avoiding**	12 (0.6)	42 (2.1)	**1,532 (76.1)**	32 (1.6)
**Abstaining**	0 (0.0)	3 (0.1)	18 (0.9)	**172 (8.5)**

* 881 cycles had a missing value from female, male or both. Trying hard as possible to conceive and trying to conceive were combined into one category. Trying hard as possible to avoid pregnancy and trying to avoid pregnancy were combined into one category. Bolded numbers indicate agreement between partners.

**Table 4:** Intercourse on a known fertile day by couple cycle pregnancy intention^*^.

Reported couple intention for cycle	Cycles with intercourse on at least one fertile day				Total number of cycles
	Known fertile days only		Known fertile days and days of undetermined fertility		Total
	N	%	N	%	
Trying hard as possible to conceive	24	80.0%	26	86.7%	30
Conceive	43	91.5%	43	91.5%	47
Unsure	78	71.6%	82	75.2%	109
Avoid pregnancy	366	38.0%	477	49.5%	963
Trying hard as possible to avoid pregnancy	202	29.0%	308	44.2%	697
Abstain	11	4.7%	16	6.8%	235
**Total**	724	34.8%	952	45.7%	2,081

* 813 cycles were not included in this table, because they were missing cycle intentions for both female and male, and/or cycle charting to indicate whether intercourse happened on a fertile day. If there was a difference in intention for the female and the male, the “most achieving” intention was used for this table.

Cumulative probabilities of pregnancy at 6 and 13 cycles by couple cycle intention are shown in **Table 5**, analyzed by single decrement lifetable. The number of cycles with each intention status and the number of couples contributing those cycles are also shown. Also displayed are sensitivity analyses where analyses were limited to cycle questionnaires completed in less than 11 days from the start of the cycle versus all cycle questionnaires regardless of completion time in the cycle; and excluding cycles that included any use of condom or withdrawal versus including all cycles regardless of condom or withdrawal use. Across all sensitivity analysis scenarios, cumulative 13-cycle pregnancy rates with cycle intention to conceive ranged from 88.0% to 89.8%, and cumulative pregnancy rates with cycle intention to avoid ranged from 29.1% to 35.3%. For cycles where the intention was to abstain from intercourse, the cumulative 13-cycle pregnancy rate ranged from 1.8% to 5.2%.

**Table 5:** Lifetable cumulative pregnancy rates at 13-cycles per 100 couples, by couple cycle pregnancy intention.

When cycle questionnaire completed	Condoms or withdrawal	Conceive			
		Couples, N	Cycles, N	Pregnancies, N	13-cycle cumulative pregnancy, %
**<11 days from cycle start of cycle**	**Excluded**	78	168	27	86.5%
	**Included**	90	190	32	88.3%
**All**	**Excluded**	86	197	32	88.0%
	**Included**	99	221	38	89.8%
		**All Avoiding Pregnancy**			
**<11 days from cycle start of cycle**	**Excluded**	223	1269	38	33.6%
	**Included**	254	1572	48	33.2%
**All**	**Excluded**	235	1435	44	32.4%
	**Included**	266	1804	56	32.6%
		**Trying as Hard as Possible to Avoid Pregnancy**			
**<11 days from cycle start of cycle**	**Excluded**	142	529	16	35.3%
	**Included**	161	648	18	31.2%
**All**	**Excluded**	156	608	19	31.7%
	**Included**	174	745	21	29.1%
		**Abstain**			
**<11 days from cycle start of cycle**	**Excluded**	70	183	1	1.9%*
	**Included**	75	190	1	1.8%*
**All**	**Excluded**	95	235	3	5.2%*
	**Included**	101	249	3	4.8%*

* No cycles with intention to abstain after 12 cycles

Cumulative probabilities of pregnancy at 6 and 13 cycles during different criteria for correct use to conceive and correct use to avoid pregnancy are displayed in **Table 6**, along with the number of cycles that met each set of criteria for correct use. For correct use to conceive, the highest cumulative 13-cycle pregnancy rate was 89.6%, with intercourse on peak-type mucus days, which are the days of higher fertility [10,29,30]. For correct use to avoid, cumulative 13-cycle pregnancy rates varied from 0.0% to 40.0%, depending on the definition used; for the primary definition of no intercourse on any day known to be fertile or any day of unknown fertility and the most conservative (restrictive) definition of correct use, the 13-cycle pregnancy rate was 15.6%. An important caveat is that for acts of intercourse on non-fertile days before the peak day, or on the fourth day after the peak day, the Creighton Model instructions specify that the non-fertile time is restricted to the end of the day, in order to allow for full observation of any mucus throughout the day [13]. However, the Creighton Model charts that were in use at the time of this study did not routinely record whether intercourse on that day occurred at the end of the day, or earlier in the day. Therefore, we do not have a completely satisfactory identification of whether any intercourse during the non-fertile days before the peak day, or on the fourth day after peak, fully met the instructions of the Creighton Model for correct timing of intercourse on that day to avoid pregnancy.

**Table 6:** Definitions of correct use with associated cycles, pregnancies, and cumulative pregnancy rates*.

Type of cycles	Cycle completely recorded	Satisfactory charting**	Exclude cycles with condoms, withdrawal, or nonstandard chart formats	Couples, N	Cycles, N	Pregnancies, N	13-cycle cumulative pregnancy, %
**To conceive: intercourse on any fertile day**	X			137	363	43	83.6%
	X	X		72	144	18	85.2%
	X	X	X	72	144	18	85.2%
**To conceive: intercourse on at least one peak-type mucus day**	X			99	208	31	87.6%
	X	X		56	90	14	89.6%
	X	X	X	56	90	14	89.6%
**To avoid pregnancy: no intercourse on any day known to be fertile, or of unknown fertility** ^***^	X			118	400	6	16.2%
	X	X		64	221	4	19.3%
	X	X	X	64	220	3	15.6%
**To avoid pregnancy: intercourse only on Peak+4 or later**	X			69	138	1	6.7%
	X	X		36	71	1	20.0%
	X	X	X	36	71	1	20.0%
**To avoid pregnancy: only cycles with intercourse on last non-fertile day pre-peak, and no recorded intercourse on days of fertility**	X			41	60	2	40.0%
	X	X		26	38	1	33.3%
	X	X	X	26	37	0	0.0%

* Analyses are restricted to participating couples in which one or both partners indicated on the exit questionnaire that they had recorded all acts of intercourse throughout the study, and to cycles with at least one recorded act of intercourse.

** Consistent observations, appropriate identification of the peak day, and accurate charting, as assessed by the Creighton Model teacher at followup.

*** Cycles with pregnancy and no intercourse on a fertile day may have included some cycles with intercourse before the end of the day on otherwise non-fertile days before the peak day, or on the fourth day after the peak day, because the time of day that intercourse occurs is not routinely annotated in the CrM chart. On these days of the cycle, any time before the end of the day is considered fertile according to CrM instructions.

In **Table 7**, we report the results of a multivariate Cox model for predictors of pregnancy in the CEIBA study couples. The strongest positive hazard ratios were identified for baseline male and female intention to have a child within 1 year or in 1–2 years (1.87 to 3.38 in the model with imputation for missing variables), or intention not reported; also baseline positive childbearing scores had positive hazard ratios, albeit not statistically significant. Study centers (grouped by crude pregnancy rates) did not have an appreciable independent impact on the probability of pregnancy.

**Table 7:** Discrete multivariate Cox Model for likelihood of pregnancy based on age, baseline motivations, intentions, and cycle intentions, with and without imputation of missing variables.

	HR (95%CI), with imputation	HR (95%CI), without imputation
**Female age (continuous)**	1.00 (0.94, 1.06)	0.96 (0.89, 1.03)
**Female positive childbearing motivation score (scale 1–4)**	1.17 (0.71, 1.94)	1.35 (0.75, 2.45)
**Male positive childbearing motivation score (scale 1–4)**	1.51 (0.78, 2.93)	1.34 (0.70, 2.56)
**Female negative childbearing motivation score (scale 1–4)**	1.09 (0.70, 1.69)	1.01 (0.60, 1.70)
**Male negative childbearing motivation score (scale 1–4)**	0.83 (0.54, 1.29)	0.86 (0.53, 1.41)
**Female, intended time to have child after entrance to study**		
>=2 years	Reference	Reference
1 year to <2 years	1.87 (0.92, 3.80)	1.52 (0.68, 3.41)
<1 year	1.99 (0.72, 5.54)	1.47 (0.44, 4.91)
Unknown	**2.43 (1.02, 5.80)**	**9.57 (2.25, 40.70)**
**Male, intended time to have child after entrance to study**		
>=2 years	Reference	Reference
1 year to <2 years	**2.04 (1.02, 4.06)**	**2.92 (1.29, 6.62)**
<1 year	**3.38 (1.26, 9.10)**	**4.73 (1.53, 14.65)**
Unknown	2.11 (0.95, 4.66)	**3.76 (1.24, 11.42)**
**Couple, intention at start of each cycle**		
Abstaining	Reference	Reference
Avoiding	0.74 (0.55, 0.99)	0.90 (0.67, 1.21)
Achieve/unsure	0.77 (0.54, 1.09)	0.79 (0.55, 1.15)
Unknown	0.93 (0.61, 1.44)	Not Applicable
**Study centers**		
Cluster 1 (9 centers)	Reference	Reference
Cluster 2 (6 centers)	1.10 (0.64, 1.87)	0.79 (0.43, 1.48)
Cluster 3 (2 centers)	0.87 (0.47, 1.62)	0.82 (0.41, 1.64)

In order to compare our results with prior Creighton Model studies, we also reported Creighton Model pregnancy classifications for each pregnancy, and calculated net pregnancy rates (probabilities) by multiple decrement life table, with the calculation done for 12 ordinal months, instead for 13 cycles [14]. These rates include all months of use in the denominator (including correct use to avoid, correct use to conceive, any other use) for all rates, and thus are not comparable to non CrMS studies. The number of pregnancies in each CrMS pregnancy category, and the net pregnancy rate per 100 couple years are reported in **Table 8**. These rates are very similar to those reported in prior studies of the Creighton Model [15–18]. However, the teaching-related pregnancy rate is higher than reported in prior Creighton Model studies. The achieving-related pregnancy rate is higher than that reported for the prior Creighton Model meta-analysis of 5 studies [16], but lower than reported in one of the individual prior Creighton Model studies [20,31].

**Table 8:** Creighton Model pregnancy classification, number of pregnancies in each classification, and pregnancy rate for each category per 100 couple-years, multiple decrement life table*.

Classification	N of pregnancies	per 100 couple years
Achieving-related	90	29.4
Method-related	1	0.3
Using-related	3	1.0
Teaching-related	5	1.7
Using/teaching-related	1	0.3
Unresolved	12	4.2
Non-CrM pregnancies	10	NA^**^
**Total**	122	37.1

* This life table analysis includes all months of use (correct use to avoid, correct use to conceive, and all other use) in the denominator of the life table, for comparability with prior CrMS studies. These rates are therefore not comparable to non-CrMS studies.

** Couples with non-CrMS pregnancies are not included in the life table analysis.

Specific reasons for the pregnancies being part of each Creighton Model pregnancy classification are detailed in **Table 9**. Each pregnancy had at least one reason for its classification, but could have more than one reason. The most common reason for an achieving-related pregnancy was intercourse on a known fertile day. Other factors also contributed to the achieving-related pregnancy classification for some pregnancies, including missing observations, inaccurate charting, “negotiating down” observations from fertile to non-fertile, and special occasions superseding usual intentions to avoid pregnancy. Using-related pregnancies were associated with errors in interpretation or application of the CrMS instructions. Teaching-related pregnancies were associated with inadequate teaching schedule or practices. Unresolved pregnancies had insufficient information for CrMS classification, usually because there was no pregnancy evaluation. Ten pregnancies were associated with condom use or withdrawal; these were considered to not be CrMS-related, and those couples were not included in the CrMS multiple decrement life table.

**Table 9:** Key issues and frequencies associated with classifying each pregnancy into a Creighton Model classification^*^.

Achieving-related pregnancies	Frequency	Percentage of cycles in classification
Intercourse on known fertile days based on peak-type mucus or within count of 3 of peak-type mucus	58	64%
Intercourse on known fertile days of any other type (non-peak mucus, abnormal bleeding, etc.)	10	11%
Intercourse before end of day pre-peak (anytime of day instead of end of day)	10	11%
Intercourse on known fertile day(s), but we don’t have details of the fertile day(s)	6	7%
Intercourse before end of day after Peak+4, while anticipating a possible double peak	1	1%
Intercourse before end of day on Peak+4	1	1%
Missed or incomplete observations, understood importance of complete observations	9	10%
Incomplete or inaccurate charting, knowing it would make more likely to conceive	4	4%
“Negotiated down” an observation from fertile to nonfertile	4	4%
Normally avoiding pregnancy, but had intercourse on fertile day because of special occasion, such as a honeymoon	3	3%
No chart or pregnancy evaluation, but follow-up form indicated actively trying for pregnancy	2	2%
Knew pregnancy was possible and that they were “taking a chance” in some sense, but thought it would take longer, surprised it happened so quickly	11	12%
**Total number achieving-related pregnancies**	**90**	**100%**
**Method-related pregnancies**		
Intercourse on last day before start of fertile window (last yellow stamp before mucus cycle)	1	100%
**Total number method-related pregnancies**	**1**	**100%**
**Using-related pregnancies** ^**^		
Misinterpreted a day that was fertile by CrMS instructions as non-fertile.	2	50%
Did not recognize double peak (stress cycle)	2	50%
Multiple significant errors in charting	1	25%
**Total number using-related pregnancies**	**4**	**100%**
**Teaching-related pregnancies** ^**^		
Follow-ups not scheduled at sufficiently frequently early on, so important method instructions not taught or clarified (e.g., Double Peak, nonpeak mucus, etc).	4	67%
Inadequate correction of improper charting and/or observation	2	33%
Instructions advanced more rapidly than recommended in CrMS teaching, and likely more rapidly than client could assimilate	2	33%
Teacher did not adequately clarify specific instructions.	1	17%
Yellow stamps given early and inappropriately, did not follow protocol for yellow stamps	1	17%
Follow-up form documentation substantially incomplete, suggests inadequate teaching	1	17%
**Total number teaching-related pregnancies**	**6**	**100%**
**Unresolved pregnancies**		
No pregnancy evaluation done, and insufficient other information	8	57%
Not enough information on first pregnancy evaluation, second pregnancy evaluation never done	2	14%
Don’t know whether intercourse on P + 4 was earlier in day or end of day (if end of day, would be method-related, if earlier would be achieving or using, depending on understanding)	3	21%
Not enough information to know whether client recognized the fertile day as actually fertile	3	21%
Significant teaching errors but unclear whether it contributed to the pregnancy	2	14%
Client stated they weren’t diligent about learning/using the method	1	7%
No intercourse charted	1	7%
Severe stress, unclear how it impacted cycle or use of method	1	7%
Contradictory information between different parts of the documentation available	1	7%
**Total number unresolved pregnancies**	**14**	**100%**
**Not CrMS-related pregnancies**		
used condoms on fertile day(s)	5	50%
used withdrawal on fertile day(s)	3	30%
used condoms or withdrawal or both on fertile day(s)	1	10%
used condom in cycle, not completely clear whether on fertile days	1	10%
**Total number not CrMS-related pregnancies**	**10**	**100%**

* Each pregnancy could have more than one reason for being classified in its respective Creighton Model category

** One pregnancy was classified as both using-related and teaching-related, and is included in the table in both categories

## Discussion

In this multi-center study of user couples of the Creighton Model FertilityCare System, couples were enrolled who were avoiding pregnancy at baseline; however, over half of couples desired to conceive within 2 years. In cycles with an intention to avoid pregnancy, there was nevertheless intercourse on one or more days of identified fertility, and/or undetermined fertility (29–50% of these cycles). Because not all couples recorded all intercourse (discussed further below), the proportions of cycles with intercourse on fertile days may in fact be somewhat higher than were ascertained in the study data. This resulted in a cumulative 13-cycle pregnancy rate among cycles with intention to avoid of about 33%. Among cycles with the intention to conceive, the cumulative 13-cycle pregnancy rate was about 90%.

In a multivariate Cox model, the strongest predictors of pregnancy were baseline intentions for pregnancy within 1 or 2 years, for both females and males, as well as positive childbearing motivation scores for both females and males. Cycle-level intentions had a smaller impact on the likelihood of pregnancy over the year of the study, suggesting that baseline intentions are more relevant, at least among these users of the CrMS, who were 90% first-time users, and 40% engaged to be married within 6 months. Because the Creighton Model is taught to emphasize the complete freedom of the couple to choose to use the method to either avoid or conceive at any point in time, it seems likely that some couples did not necessarily maintain their declared intention at the beginning of the cycle throughout the remainder of the cycle [11]. In other words, the fertility appreciation focus of the Creighton Model system may encourage proceptive behaviors [31–33].

Other population-based studies have found that women may have intercourse without contraception during potentially fertile days either because they don’t really think the day is fertile, they don’t really mind if they get pregnant, or their partner doesn’t want to use any contraceptive [34]. This seems partially similar to some of the couples in this study, some of whom reported being surprised that pregnancy happened so quickly.

In order to compare the CEIBA data to prior CrMS studies, we also evaluated all pregnancies according to the Creighton Model pregnancy classification system, and calculated multiple decrement life table cumulative pregnancy rates [14]. We identified factors that were associated with each pregnancy classification. Although the pregnancy evaluations assess the dynamics of use only among those who conceive (i.e., there is no comparable evaluation for those who do not conceive), they do provide insight into what factors are associated with conceiving during use of the Creighton Model FertilityCare System, detailed in **Table 9**. The majority, 90/122 (74%) of pregnancies were classified as achieving-related. Intercourse on known fertile days accounted for the majority of achieving-related pregnancies. A number of other behaviors listed in **Table 9**, including intercourse before the end of the day in the prepeak or preovulatory non-fertile time, inadequate observation, or incomplete charting are routinely identified as such by CrMS teachers during follow-up sessions as “achieving-related,” where they inform the couples that such behaviors are likely to result in pregnancy. For CrMS avoiding-related pregnancies, related factors included errors in application of the instructions, and/or inadequate teaching practices contributed to the occurrence of some pregnancies.

The Creighton Model multiple decrement life table pregnancy rates are not comparable to studies of other methods of family planning, because the denominator includes all months for all the cumulative pregnancy rates. For example, the denominator for the method-related pregnancy rate includes all months of couple time in the study, including many months of achieving-related use, which artificially lowers the method-related pregnancy rate [24]. To compare method effectiveness between studies, the correct use pregnancy rate (often called the “perfect use” pregnancy rate), is an appropriate measure [22]. We therefore calculated correct use pregnancy rates for conceiving, as well as for avoiding pregnancy. For the same reason, CrMS using-related pregnancy rates, which are based on a denominator of all months (or cycles), are not comparable to the pregnancy rates during only the cycles with intention to avoid pregnancy.

In the analyses of correct use to conceive or to avoid pregnancy, the 13-cycle cumulative pregnancy rates were 89.6% and 15.6%, respectively. However, there were important limitations in the correct use analyses. First, 112 couples could not be included in the analyses, because we could not be confident that all intercourse was recorded. Those included in the correct use analyses were the couples (n = 184) who indicated on the exit questionnaire that they had recorded all intercourse during the study all of the time without exception, or nearly all of the time with maybe a rare exception. Of the remaining couples, 36 responded that they did not record all intercourse, and 76 did not have any data for this question. Recent studies of some FABMs have found many persons do not record intercourse consistently [35,36], suggesting a need to be attentive and vigilant about whether intercourse has been adequately recorded to conduct analyses of correct use [24,37].

A second limitation of the correct use analyses is that instructions of the Creighton Model to avoid pregnancy on non-fertile days before the peak day, or on the fourth day after peak day, are to restrict the timing of intercourse to the end of day, in order to allow any mucus that may have been produced at the cervix time to travel through the vagina and be sensed at the vulva [13]. Therefore, any time earlier in the day on these days is considered to be a time of potential fertility for intercourse, even if no mucus is identified at the end of the day. During the time of this study, the Creighton Model chart did not have a standardized way to record whether intercourse occurred before the end of the day, or at the end of the day. Therefore, we can’t exclude the possibility that some acts of intercourse on non-fertile days before the peak, or on the fourth day after peak may have in fact occurred earlier in the day. This possibility is queried during pregnancy evaluations, but not necessarily for cycles that did not result in conception. After this study was completed, the CrMS now has a mobile app accessible version that routinely records whether intercourse was at the end of the day, or before the end of the day, so this information should be available for future analyses of correct use of the CrMS to avoid pregnancy.

Pregnancy that occurs from intercourse outside the identified fertile window (with all intercourse recorded) could occur from two possible scenarios: 1) after the identified day of ovulation, i.e., at the end of the fertile window, or 2) before the identified day of ovulation, i.e., at the beginning of the fertile window [10,28]. Within the CEIBA data, we explored both of these possibilities in alternate definitions of correct use (postpeak intercourse only, or intercourse on the “last non-fertile day” before peak day. These analyses yielded 13-cycle cumulative pregnancy rates of 0% to 40%. The wide range of estimates reflects the very limited sample size of cycles that met the criteria for these analyses. Therefore, we consider the results of these analyses exploratory, and not reliable for actual estimates of correct use in the respective time windows before or after the identified fertile window.

We conducted sensitivity analyses for the impact of the use of condoms or withdrawal for all of the analyses of cumulative pregnancy rates. Overall, there was little impact on the pregnancy rates, whether cycles with condom or withdrawal use were included or excluded. Other studies of natural family planning methods used to avoid pregnancy have found somewhat lower pregnancy rates with abstinence only, as compared with use of barrier methods at the fertile times [38–40].

Most studies of natural family planning or fertility awareness-based methods have focused on use only to avoid pregnancy [4,41], while relatively fewer have focused only on use to conceive [1]. The Creighton Model FertilityCare System is taught as a complete family planning and fertility appreciation system, available to couples for avoiding pregnancy or conceiving, at any point of time [11]. We therefore examined and reported all use of the CrMS, and all pregnancies in this study. Reporting on all pregnancies, inclusive of use intended to avoid pregnancy and use intended to conceive, has been recommended as one of the criteria for high quality studies of natural family planning or fertility awareness-based methods [4].

In summary, this study of the Creighton Model FertilityCare System among couples with high baseline motivation for childbearing and a large proportion with intention to conceive in the coming 1–2 years, found relatively high pregnancy rates overall, including during cycles with stated intention to avoid pregnancy, and in analyses of correct use to avoid pregnancy. In nearly half of cycles with stated intention to avoid pregnancy, the couple had intercourse on fertile days or days of unknown fertility. Most pregnancies (90/122) were classified as related to achieving-related behaviors, a spectrum of behaviors that CrMS teachers identify and teach during follow-up sessions as being likely to result in pregnancy. The pregnancy evaluations provide important insight into factors related to conceiving during use of a natural family planning or fertility appreciation method.

## Supporting information

S1 Text Creighton Model FertilityCare System (CrMS) chart details; Identification of potentially fertile days, non-fertile days, and days of unknown fertility; Ascertainment of use of condoms or withdrawal. (DOCX)
