# Supplementary material for: Pregnancies, intentions, and fertility behaviors during use of the Creighton Model FertilityCare System after initial intention to avoid pregnancy: Results from the Creighton Model effectiveness, intentions, behaviors assessment study
Source: PLoS One. 2025 Jul 29;20(7):e0328806. doi: 10.1371/journal.pone.0328806 (PMC12306780; doi:10.1371/journal.pone.0328806)
Supplement: S1 Text — (DOCX) [file pone.0328806.s001.docx]

**S1 Text**

**Creighton Model Fertility*Care* System (CrMS) chart details; Identification of potentially fertile days, non-fertile days, and days of unknown fertility; Ascertainment of use of condoms or withdrawal**

CrMS chart details

In the standard teaching of the CrMS, couples are taught track and record observations of vaginal bleeding, and vaginal discharge (including cervical mucus) on a daily basis on the CrMS chart (a daily diary), using the standardized vaginal discharge recording system (VDRS) (Hilgers, 2004). The woman is instructed to use plain toilet tissue, wipe from front to back across the vaginal opening, and finger test any vaginal discharge. The VDRS contains codes for the stretch and color observed, and for the sensation of lubrication while wiping. Each day, a stamp is placed by the couple to interpret the likelihood of pregnancy, or “fertility” of that day. Plain green stamps and plain yellow stamps indicate days of “infertility,” baby stamps (which can be white, green, or yellow) indicate days of “fertility,” and red stamps indicate days of bleeding, which can be “fertile” or “infertile,” depending on whether they are part of the menstrual flow, and also any concurrent mucus observations. The couple also records whether intercourse or genital contact occurred on each day. During follow-up sessions with the CrMS teacher, the teacher reviews the couples recording of the VDRS and placement of the stamps, and corrects them as necessary. The couple’s personal CrMS chart was copied and abstracted for this study, which was used to interpret each day as fertile or non-fertile (as described below), and the timing of acts of sexual intercourse on fertile or non-fertile days of the cycle.

During the time period of this study, all CrMS charts were kept on paper (i.e. the CrMS mobile web app had not yet been developed). However, some participants devised their own version of the CrMS chart, usually with an excel spreadsheet. We noted these type of charts as “non-standard” CrMS charts.

Identification of potentially fertile days, non-fertile days, and days of unknown fertility

Each day was designated as potentially fertile if it met one or more of the following criteria:

- any “peak” fertility day according to CrMS instructions, which includes any of the following circumstances(Hilgers et al., 2002)
  - the last consecutive day with any mucus that is clear, partly clear, stretchy (more than one inch), or lubricative (any one of these characteristics)
  - the last of any series of at least three consecutive days with any kind of mucus that is not clear, partly clear, stretchy (more than one inch), or lubricative
  - a day of vaginal bleeding that is not part of the menstruation days
  - any day with incomplete or uncertain observations
  - Note: a cycle may have multiple “peak” fertility days; only one of them will correlate to the day of ovulation (Stanford et al., 2020).
- the three days following a CrMS peak fertility day
- final stamp (after any teacher corrections) with a baby (white baby, green baby, yellow baby)
- red stamp (indicating bleeding) with any mucus qualities of any type recorded (other than “dry” descriptions indicating no mucus discharge that day)
- any day without a stamp that had any mucus qualities of any type recorded (other than “dry” observations).

Each day was designated as non-fertile if it did not meet any of the previous criteria for a fertile day, and it also had a final stamp (after any teacher correction) of plain green or plain yellow, or it had a red stamp with “dry” mucus observations, or it had a red stamp with moderate or heavy menstrual bleeding and absent mucus observations.

Days that did not meet any of the above criteria (such as missing stamps and missing observations), were considered to have unknown fertility status for our analysis. In addition, each of the three days following a day of unknown fertility (i.e., within a count of three after a day of unknown fertility) was considered to also be a day of unknown (potential) fertility, unless it was already a fertile day by the other criteria described above.

In the analysis of correct use to conceive, the couple needed to have intercourse on at least one known fertile day. In the analyses of correct use to avoid pregnancy, days with unknown fertility status were classified as potentially fertile days; therefore, intercourse on a day of unknown fertility, or a day of fertility, would be inconsistent with correct use to avoid pregnancy. This is consistent with the use of the CrMS: CrMS teachers instruct users to treat days with missing observations, and the following three days, as fertile days.

Ascertainment of use of condoms or withdrawal

We identified cycles where condoms or withdrawal had been used by any one of three ways: 1) cycles in which the couple had noted on their CrMS chart the use of one or both methods, 2) responses to the start of cycle (SOC) questionnaire by either partner indicating condom or withdrawal use in the prior cycle, and/or 3) affirmative responses to questions from the CrMS teacher about the use of barrier methods, which are a routine part of the follow-up form used by the CrMS teacher to guide the follow-up session. As part of the study, couples were asked to indicate any use of condoms or withdrawal on their CrMS chart.

Creighton Model pregnancy classification

We encouraged every participant who became pregnant to meet with their CrMS teacher to complete a pregnancy evaluation. During this study, there were two versions of the standard CrMS pregnancy evaluation: a short form and a long form (with more detail); for this study, we requested that the long form always be used. The CrMS pregnancy evaluation was usually conducted by the couple’s own CrMS teacher, but in a few cases, at the request of the couple, we arranged for it to be conducted by a different CrMS teacher. One major purpose of the CrMS pregnancy evaluation was to assign the pregnancy into one of the CrMS pregnancy classifications, as defined in past CrMS research and described below. As per standard CrMS protocol, if the pregnancy classification was unresolved or method-related, a second pregnancy evaluation was conducted by a different CrMS teacher to corroborate or update the classification.

For the purposes of this study, all pregnancies were also reviewed again by an expert panel of CrMS educators and a CrMS medical consultant (K.D. Daly, K. Rivet, and J.B. Stanford), to assign each pregnancy a final CrMS pregnancy category for the purposes of this study. This review was based on the written records of the CrMS pregnancy evaluation, the CrMS chart, the CrMS follow-up form, and in a few cases, additional communication with the teacher(s) who had taught the couple. The pregnancy classification assigned in this final expert review was used in the analyses reported in this paper. We used the standard CrMS pregnancy classification system, listed below. This classification is not based on a couple’s stated intentions, but rather on information about understanding of fertility (identification by the couple of fertile and non-fertile days) and behavior in the actual use of the method (including timing of intercourse, and also consistency in observing, charting, and interpreting the biomarkers of fertility according to CrMS instructions) (Hilgers, 1984, Hilgers and Stanford, 1998).

Creighton Model pregnancy categories

The CrMS pregnancy classification categories are as follows (Hilgers et al., 2003):

I. **Achieving related pregnancy**. From the available information, the method was used as a system to achieve pregnancy, and the couple became pregnant.

II. **Avoiding related pregnancy**. From the information available, the system was used as a system to avoid pregnancy, and the couple became pregnant.

[This category has the following subcategories:]

1. **Method related**. From the available information, the system was **used correctly** as a system to avoid pregnancy, and the couple became pregnant.
2. **Using related**. From the available information, the method was **used incorrectly** (but taught correctly) as a system to avoid pregnancy, and the couple became pregnant.
3. **Teaching related**. From the available information, the method was **taught incorrectly** (but used correctly according to instruction) as a system to avoid pregnancy, and the couple became pregnant.
4. **Using/teaching related**. A combination of using related and teaching related.

III. **Unresolved pregnancy**. From the available information, the circumstances of the pregnancy cannot be placed into any of the above classifications.

IV. **Not CrMS-related**. [Pregnancies that occurred while also using other methods of family planning, e.g., condoms or withdrawal]

References for Supplemental Material

Hilgers TW. The statistical evaluation of natural methods of family planning. *International review of natural family planning* 1984;8: 226-264.

Hilgers TW. Basic charting and chart reading. In Hilgers TW (ed) *The medical and surgical practice of NaProTechnology*. 2004. Pope Paul VI Institute Press, Omaha, pp. 107-118.

Hilgers TW, Daly KD, Hilgers SK, Prebil AM. *Creighton Model Fertility Care System: A standardized, case management appproach to teaching, Book 1.* 2nd edn, 2002. Pope Paul VI Institute Press, Omaha, NE.

Hilgers TW, Hilgers SK, Prebil AM, Daly KD. *Creighton model fertilitycare system: a standardized, case management approach to teaching-book 2: advanced teaching skills*, 2003. Pope Paul VI Institute Press, Omaha, NE.

Hilgers TW, Stanford JB. Creighton Model NaProEducation Technology for avoiding pregnancy. Use effectiveness. *J Reprod Med* 1998;43: 495-502.

Stanford JB, Schliep KC, Chang CP, O'Sullivan JP, Porucznik CA. Comparison of woman-picked, expert-picked, and computer-picked Peak Day of cervical mucus with blinded urine luteinising hormone surge for concurrent identification of ovulation. *Paediatr Perinat Epidemiol* 2020;34: 105-113.
